# A graphical simulation software for instruction in cardiovascular mechanics physiology

**DOI:** 10.1186/1475-925X-10-8

**Published:** 2011-01-25

**Authors:** Reto A Wildhaber, François Verrey, Roland H Wenger

**Affiliations:** 1 Institute of Physiology and Zürich Center for Integrative Human Physiology (ZIHP), University of Zürich, Winterthurerstrasse 190, CH-8057 Zürich, Switzerland

## Abstract

**Background:**

Computer supported, interactive e-learning systems are widely used in the teaching of physiology. However, the currently available complimentary software tools in the field of the physiology of cardiovascular mechanics have not yet been adapted to the latest systems software. Therefore, a simple-to-use replacement for undergraduate and graduate students' education was needed, including an up-to-date graphical software that is validated and field-tested.

**Methods:**

Software compatible to Windows, based on modified versions of existing mathematical algorithms, has been newly developed. Testing was performed during a full term of physiological lecturing to medical and biology students.

**Results:**

The newly developed CLabUZH software models a reduced human cardiovascular loop containing all basic compartments: an isolated heart including an artificial electrical stimulator, main vessels and the peripheral resistive components. Students can alter several physiological parameters interactively. The resulting output variables are printed in x-y diagrams and in addition shown in an animated, graphical model. CLabUZH offers insight into the relations of volume, pressure and time dependency in the circulation and their correlation to the electrocardiogram (ECG). Established mechanisms such as the Frank-Starling Law or the Windkessel Effect are considered in this model. The CLabUZH software is self-contained with no extra installation required and runs on most of today's personal computer systems.

**Conclusions:**

CLabUZH is a user-friendly interactive computer programme that has proved to be useful in teaching the basic physiological principles of heart mechanics.

## Background

The principles of cardiovascular mechanics are complex and due to their dynamic nature, are not readily comprehensible by studying static textbooks. Therefore, the visualization of the dynamic processes by e-learning tools which allow the user to vary the physiological variables, provides a useful tool for teaching students of medicine and biology. However, the currently available Software Laboratory [1] is more than twenty years old and can no longer be maintained on the latest systems software. A new programme that allows students to verify interactively the theories from the lectures was therefore necessary. This software was to be easy-to-use, small and self-contained, allowing efficient implementation, spreading and installed simply into the students' various personal computer systems.

Internet queries, including the Medline database, revealed a number of related educational tools. However, none of them satisfactorily met our graphical and didactical needs. Basically, the new tool should teach human cardiovascular mechanics and focus on the healthy adult cardiovascular system. The intention is to give students a degree of freedom in setting the model's input parameters, while the output parameters are preferably shown graphically.

## Methods

### Software development

Two established mathematical models were modified to generate the new CLabUZH software. The Isolated Heart Laboratory (IHL) and the Closed Circular Laboratory (CCL). The IHL model was developed by Sagawa [2] and Suga [3] and adapted by Peterson [1]. The model for CCL is based on the algorithm developed by Peterson and Campbell. The Cardiovascular Function Labs (CFL) programme, containing both algorithms, was published in 2003 by Crawford (School of Medical Sciences, University of New South Wales, Australia) in co-operation with Peterson [1] and was provided under GNU General Public License version 2 (GPL2, http://www.gnu.org) in 2009. Code fractions, mainly the model's basic algorithm, were taken from the CFL software code and reused in a modified form in CLabUZH. CLabUZH is written in the programming language JAVA 2 (Sun Microsystems, http://www.sun.com) and is distributed free of charge under GPL2.

### Software assessment

The CLabUZH software was assessed and improved during the practical courses in physiology for second year medical students and fourth year human biology students, numbering approx. 270 and 40 students, respectively, at the University of Zürich, Switzerland (UZH) during one semester in 2009.

## Results and Discussion

### Mathematical features of the existing software

Both existing IHL and CCL models simulate an isolated canine heart [1-3]. The nervous and humoral sympathetic and parasympathetic regulation is absent. The lack of nervous innervations is compensated by an artificial pacemaker, working at a fixed rate. The models were designed as a recursive mathematical algorithm working at discrete time intervals. Pressure, volume and flow values of the cardiovascular system are continuously re-calculated on the basis of the previous values with a time interval of 1 ms.

The IHL model uses the left heart chambers only and is connected to an unlimited virtual blood source, providing a constant preload pressure [1]. The model is suitable for the study of the basic heart cycle covering the ventricular volume, pressure and timing relations and the valve mechanism. Primary effects such as the Frank-Starling Law - described as "the larger the diastolic volume of the heart (...) the greater is the energy of its contraction" [4] - or the blood pressure dumping effect of the arterial system, also known as Windkessel Effect, can all be examined.

In the CCL model, all four heart chambers are used. They are fully connected to a cardiovascular circulation loop consisting of both the systemic and the pulmonary circulation. This setting was designed to study the interaction of the two circulatory loops. The contractility of the left and right ventricles can be regulated separately. The model is useful as a means of exploring the systemic effects of various pathological conditions, e.g. partial heart insufficiencies.

### Mathematical features of the new CLabUZH software

The two existing algorithms underwent three major changes: I) All internal canine parameters were changed to human values. Average values for cardiovascular healthy male and female individuals were taken [5]. II) The missing atria were added to the heart model as they contribute a considerable fraction of the total cardiac output under conditions of an increased heart rate. Atrial contraction increases the preload and thereby contributes to the cardiac output by up to 10% [6]. III) An electrical heart stimulator was added. The electrical stimulator contains a dynamic electrocardiogram (ECG) generator and triggers the mechanical heart activity. This ECG generator was implemented as an independent software module, concomitantly synthesizing the single ECG curve just-in-time. It draws on a library of records of ECG fractions, originating from an arbitrarily chosen single subject with a healthy cardiovascular system. The fractions are reassembled by the generator in respect to correct timing and currently selected heart rate. The timing is approximated by a set of mathematical second order terms according to Shorten and Burke [7]. This results in a dynamically-adjusted time delay between the electrical and mechanical cardiac cycle and produces a heart rate dependent diastolic time fraction (DHF) which has a major impact on the myocardial perfusion [8].

### Graphical features of the new CLabUZH software

**Figure 1:**
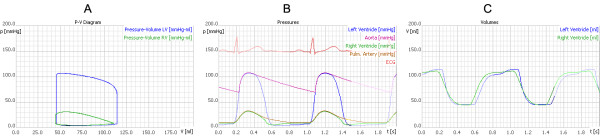
**X-y diagrams of the CCL Model output variables of the CLabUZH software**. All three diagrams are continuously updated with volume, pressure and electrocardiogram data. A cross hairs cursor allows accurate reading of data values. Pressure-volume loop (A), pressure of heart and vessel compartments (B), and ventricle volumes (C) are displayed.

The software code for CLabUZH has been written using the Java 2 programming language and applies the Model-View-Control principles [9]. This software design pattern keeps the model, including the algorithm, strictly separate from the graphical and user interaction components. CLabUZH is designed for graphical window-based computer application. Several physiological parameters can be altered by the students. The resulting output variables are printed in x-y diagrams and visualized additionally in an animated graphical model. The main window has a toolbar located at the top, with buttons to switch between the models, to throttle simulation speed or temporarily to halt the execution. The main area below the toolbar is subdivided horizontally into two sections. The upper subdivision contains three x-y diagrams (Figure 1A-C), continuously recording cardiac pressure and volume data. The new data are superimposed on the preceding data, which fade into the background.

Furthermore, a single, not scaled electrocardiogram (ECG) from the generator is recorded in the centre diagram. Each curve on the diagrams can be concealed or made visible by clicking the relevant rubric in the legend. Moving the mouse over one of the diagrams will allow the cursor to alter to a cross hairs one and so facilitate accurate reading of data values or alternatively provide differential measurements when the mouse is dragged. For didactical reasons, the scale of all diagrams is kept fixed.

**Figure 2:**
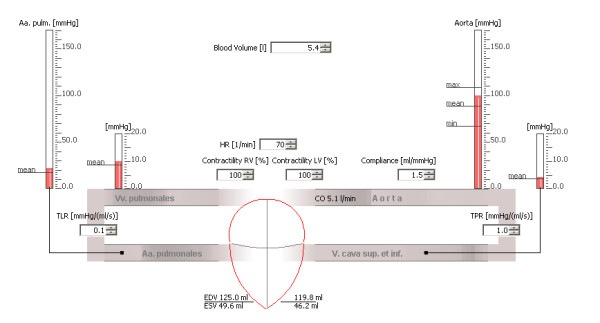
**An animated graphical view of the closed circulation loop (CCL model) of the CLabUZH software, including the listboxes controlling the input parameters and the manometers displaying current pressure values**. Abbreviations: arteriae pulmonales, Aa. pulm.; heart rate, HR; left ventricle, LV; right ventricle, RV; end-diastolic volume, EDV; end-systolic volume, ESV; total peripheral resistance, TPR; total lung resistance, TLR.

The lower window subdivision contains a dynamic graphical view of the currently selected model (Figure 2). Multiple listboxes are integrated into this view. Each listbox controls one of the input parameters of the model and can be changed manually. Changes will take effect instantaneously and run immediately into the model's calculations. A comprehensive list of the input parameters available, including currently valid value ranges, for both CFL and IHL models is shown in Table 1.

**Table 1:** Input parameters available in the IHL and the CCL model of the new CLabUZH software

Parameter	Definition	Default value	Limits (min/max)	Model
CVP	Venous blood pressure in the vena cava	10 mmHg	0/30 mmHg	IHL

HR	Heart rate	70 min^-1^	40/160 min^-1^	IHL/CCL

Contractility LV/RV	Myocardial contractility of the left (LV) or right ventricle (RV) rated relative to an average inotropy.	100%	25/200%	IHL/CCL

TPR	Total peripheral resistance	1.0 mmHg (ml s^-1^)^-1^	0.2/2.0 mmHg (ml s^-1^)^-1^	IHL/CCL

Compliance	Rated aorta wall elasticity, defined as (δvolume/δpressure).	1.5 ml mmHg^-1^	0.5/2.0 ml mmHg^-1^	IHL/CCL

TLR	Total lung resistance	0.1 mmHg (ml s^-1^)^-1^	0.02/0.5 mmHg (ml s^-1^)^-1^	CCL

Blood volume	Total blood volume in the circulation	5.2 l	3.0/6.4 l	CCL

The graphical view includes an animated heart placed at the centre of the screen with other elements of the circulation loop grouped around it. The animated heart displays a human heart in an abstract four chamber view. Its shape is divided into the four compartments. The horizontal division corresponds to the atrioventricular plane, the vertical division to the ventricle septum (Figure 2). The resulting volume of each chamber is in proportion to the current chamber fillings. The reduction in volume during the contraction phase mainly results in a shortening of the longitudinal axis and to a lesser extent in the radial contraction. This distribution between the axes is based on the findings reported by Carlssen et al [10]. In conjunction with the atrioventricular conduction delay, a considerable atrioventricular plane displacement (AVPD) results. This AVPD covers up to 60% of the stroke volume of the left ventricle [11].

The other elements of the circulation loop in the graphical view, such as the arterial and venous vessels, are shown and animated according to their instantaneous blood pressure. To demonstrate pulse propagation the vessels inflate and deflate in rhythm with every stroke.

### Systems software compatibility to the new CLabUZH software

CLabUZH runs on the majority of today's personal computers and has been tested on the current versions of Microsoft Windows (Windows XP Professional SP2, Windows Vista Home Premium), Linux and Mac's operating systems (OS X v10.5). CLabUZH does not need an installation programme and will not store any configuration data in the PC. Furthermore, it runs directly from read-only or removable media. For convenient installation in large-scale computer training rooms, it can also be operated directly from network drives.

### Assessment of the new CLabUZH software in practical student's courses

**Figure 3:**
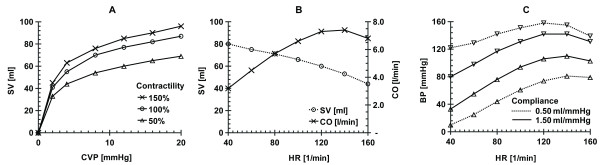
**Examples of results obtained by students after working through the training instructions with the IHL model and using the default parameter settings**. (A) Non-linear relationship between stroke volume (SV), central venous pressure (CVP) and contractility rates at a constant heart rate (HR) of 70 min^-1^; (B) Relationship between SV, cardiac output (CO) and HR at a constant CVP of 10 mmHg in absence of sympathetic stimulation of the isolated heart; (C) Change in systolic (downwards pointing triangle) and diastolic (upwards pointing triangle) blood pressure (BP) with increasing heart rate for arterial vessels with normal compliance (1.5 ml/mmHg) and reduced compliance (0.5 ml/mmHg)..

To test the functionality of CLabUZH software, it was used for a full term of instruction in cardiovascular mechanics, comprising in total over 300 participants. The use of the software was closely followed and observations made immediately applied for the improvement of the software algorithms, graphical display, interface, and the training instructions. Figure 3 shows examples of the results of single tasks. It should be noted that the CLabUZH software simulates an isolated heart without the autonomic nervous system. Especially at higher heart rates the difference to an *in vivo *setting becomes evident and needs manual compensation if *in vivo *results are demanded. All students were able to handle the software after a brief introduction and the majority of the exercises were completed within the anticipated 3 to 4 hours.

### Limitations of the new CLabUZH software

Some restrictions caused by the limitations of the mathematical algorithm should be mentioned: the algorithms are based on a data matrix of volume, pressure and flow speed values and the circulation loop is subdivided into a limited number of compartments (5 compartments for IHL and 10 for CCL). Blood-mass and the resulting inertia have not been taken into consideration. Thus, an aberration must be taken into account especially during high energy ejection phases and in simulation of the pulse propagation. Nevertheless, the results of the algorithms are satisfactory and conform to our requirements.

## Conclusions

We have found the CLabUZH software to be a solid and comprehensive educational tool for the teaching of heart mechanics. The software is rapidly installed, requires very little maintenance and is easy to use, even by persons with limited computer skills. The current software version is 8.4.4 (Additional file 1). The latest software version is also available to download from: http://www.physiol.uzh.ch/teaching.html

## Competing interests

The authors declare that they have no competing interests.

## Authors' contributions

The software development was done by RAW. All authors participated in the design and optimization of the software and they drafted the manuscript. All authors read and approved the final manuscript.

## Supplementary Material

Additional file 1 **CLabUZH_844.jar**. CLabUZH software that models a reduced human cardiovascular loop. Click here for file
